# Silencing and Nuclear Repositioning of the *λ5* Gene Locus at the Pre-B Cell Stage Requires Aiolos and OBF-1

**DOI:** 10.1371/journal.pone.0003568

**Published:** 2008-10-30

**Authors:** Alexander Karnowski, Chun Cao, Gabriele Matthias, Sebastian Carotta, Lynn M. Corcoran, Inga-Lill Martensson, Jane A. Skok, Patrick Matthias

**Affiliations:** 1 Friedrich Miescher Institute for Biomedical Research, Novartis Research Foundation, Basel, Switzerland; 2 The Walter and Eliza Hall Institute of Medical Research, Parkville, Victoria, Australia; 3 Laboratory of Lymphocyte Signaling and Development, The Babraham Institute, Cambridge, United Kingdom; 4 Department of Immunology and Molecular Pathology, University College London, London, United Kingdom; 5 New York University School of Medicine, New York, New York, United States of America; National Institute on Aging, United States of America

## Abstract

The chromatin regulator Aiolos and the transcriptional coactivator OBF-1 have been implicated in regulating aspects of B cell maturation and activation. Mice lacking either of these factors have a largely normal early B cell development. However, when both factors are eliminated simultaneously a block is uncovered at the transition between pre-B and immature B cells, indicating that these proteins exert a critical function in developing B lymphocytes. In mice deficient for Aiolos and OBF-1, the numbers of immature B cells are reduced, small pre-BII cells are increased and a significant impairment in immunoglobulin light chain DNA rearrangement is observed. We identified genes whose expression is deregulated in the pre-B cell compartment of these mice. In particular, we found that components of the pre-BCR, such as the surrogate light chain genes *λ5* and *VpreB*, fail to be efficiently silenced in double-mutant mice. Strikingly, developmentally regulated nuclear repositioning of the *λ5* gene is impaired in pre-B cells lacking OBF-1 and Aiolos. These studies uncover a novel role for OBF-1 and Aiolos in controlling the transcription and nuclear organization of genes involved in pre-BCR function.

## Introduction

The developmental fate of B lymphocytes is tightly controlled by a large number of transcription factors [1]–[3]. The differential expression of factors like PU.1, Ikaros, ID2, Notch-1, E2A, EBF and PAX5 controls the progressive commitment of pluripotent hematopoietic stem cells to the lymphoid and then to the B cell lineage. These and other factors orchestrate the transcriptional program required for proper developmental progression along the B cell pathway. Rearrangement of the immunoglobulin heavy chain (IgH: μHC) takes place at the pre-BI cell stage and its functionality is checked at the subsequent pre-BII cell stage [4], [5]. At this stage, the heavy chain associates with the surrogate light chain (SLC), encoded by the *VpreB* and *λ5* genes, to form the pre-B cell receptor (pre-BCR), which is displayed at the cell surface. This represents a first checkpoint in early B cell development. Signaling by the pre-BCR induces a proliferative burst and cell survival, followed by downregulation of the *VpreB* and *λ5* genes, exit from the cell cycle and induction of immunoglobulin light chain rearrangement (IgL: κ or λ) in small pre-BII cells [6]. Surface expression of a functional B cell receptor (BCR), consisting of IgH paired with IgL, is essential for progression through the second checkpoint. The immature B cells that pass this selection exit the bone marrow and migrate to the spleen where they continue their differentiation through several transitional B cell stages [7], [8], which undergo negative selection processes [9]. A small number of the surviving cells, possibly having a lower level of BCR signaling [10], differentiate into the naïve and sessile marginal zone B (MZB) cells, while the majority of the surviving transitional B cells become naïve follicular B cells. These long-lived cells circulate through the follicles of the spleen, lymph nodes and the bone marrow.

The transcriptional coactivator OBF-1 (Bob-1, Oca-B) is essential in late B cell development. OBF-1 is predominantly expressed in B lymphocytes and can form ternary complexes on permissive octamer sites with the POU domain transcription factors Oct1 and/or Oct2 [11]–[13]. Work from several laboratories has shown that the deletion of OBF-1 leads to a reduction in the newly arriving transitional B cells in the spleen and to diminished numbers of recirculating B lymphocytes in the bone marrow [14], [15]. Furthermore, OBF-1 mutant mice have a severely impaired T cell dependent (TD) humoral immune response and fail to form germinal centers (GC) [16], [17]. The absence of GCs may at least in part be due to the reduced expression of the Ets factor Spi-B in *OBF-1* ^−/−^ B cells [18]. In addition, the reduced humoral immune response correlates with the requirement of OBF-1 for formation of antibody secreting cells [19]. OBF-1 is also required for a normal proliferative and signaling response to BCR stimulation [20], [21], and in a *C57BL/6* genetic background, it is also critical for the development of MZ B cells [22].

The zinc-finger transcription factor Aiolos is expressed in early B and T cell subsets as well as in mature B cells [23]. It can form heterodimers with Ikaros and it activates or represses genes by recruiting chromatin remodeling complexes [24]–[26]. Ablation of Aiolos results in a phenotype that is in certain aspects the opposite of what is observed in *OBF-1^−/−^* mice: a slight increase in pre-B cells in the bone marrow, reduction of peritoneal B1 B cells and most notably constitutive formation of GCs [10], [27]. Aiolos deficient B cells show an enhanced proliferative and signaling response to BCR stimulation, which may at least partly explain the spontaneous formation of GCs and the development of autoantibodies in Aiolos deficient mice [21], [27]. In mice lacking Aiolos, anti-DNA autoantibodies accumulate as immune complexes in the kidney, which can result in signs of renal failure and symptoms resembling those found in human systemic lupus erythematosus (SLE) [21].

In contrast to the late developmental defects seen in the single-mutants, the combined loss of Aiolos and OBF-1 has a strong impact on early B cell development and results in a severe reduction of the immature B cell pool in the bone marrow. The effect on early B cell development is accompanied by a reduction in the numbers of peripheral mature B cells and an absence of the SLE-like symptoms [21]. The cellular and molecular mechanisms underlying this phenotype are not well understood. Here we have examined the synergistic role of Aiolos and OBF-1 within the regulatory network that control early B cell development. For this, pre-BII cells and their transcriptomes were characterized from single-and double-mutant mice. RNA expression profiling revealed that the genes encoding the SLC fail to be properly downregulated in small pre-BII cells from Aiolos/OBF-1 double-deficient mice. Moreover, three-dimensional DNA fluorescence *in situ* hybridization (3D FISH) analysis demonstrated that the subnuclear organization of the *λ5* locus and its correct relocalization during B cell development strongly depend on Aiolos and moderately on OBF-1.

## Materials and Methods

### Mice and cells

The *Aiolos^−/−^* and *OBF-1^−/−^* mice have been described previously [16], [27]. Animal experimentation was carried out according to regulations effective in the Kanton of Basel-Stadt, Switzerland as well as in accordance with the FMI internal regulations under supervision of the FMI Animal Committee. The mice were housed in groups of one to six animals at 25°C with a 12∶12 h light-dark cycle. They were fed a standard laboratory diet containing 0.8% phosphorus and 1.1% calcium (NAFAG 890, Kliba, Basel, Switzerland). Food and water was provided *ad libitum*. All mice were analyzed between 6 and 15 weeks of age. A20J is a murine B cell lymphoma cell. Cells were cultured in RPMI 1640 supplemented with 10% fetal calf serum, 2 mM glutamine, 100 mg/ml penicillin, 100 mg/ml streptomycin, and 50 mM 2-mercaptoethanol.

### Antibodies and Flow Cytometry

Single cell suspensions of purified bone marrow cells or splenocytes were stained with fluorochrome or biotin-labeled antibodies. Cells were analyzed on a FACSCalibur (BD Immunocytometry Systems) using a lymphocyte live gate. Cell sorting was carried out on a MoFLOW cell sorter (Dako-Cytomation). Data were analyzed with CellQuest (BD Immunocytometry Systems) and FlowJo (Tree Star) software. For intracellular stainings, cells were fixed for 10 minutes with 2% paraformaldehyde/PBS and permeabilized with 0.1% saponin/PBS. At least 1×10^6^ cells were stained with the following antibodies: anti-B220-APC (RA3-6B2); anti-CD25-Biotin or PE (7D4, BD Pharmingen), anti-ckit PE (ACK4, BD Pharmingen), anti-IgM-FITC (1B4B1, Southern Biotech); anti-IgD-Biotin (I-19); anti-pre-BCR-Biotin (SL156); anti-λ5-Biotin (LM34). For secondary stainings the following strepdavidine conjugates were used: strepdavidine-PECy7 (eBiosciences); streptavidine-PECy5.5 (Caltag).

### Real time RT-PCR analysis

Bone marrow or splenic cells from 3∼5 age-matched mice were pooled and sorted. RNA was extracted from 4×10^4^ to 1×10^6^ primary cells or cell lines, using TRIzol or the AbsolutelyRNA micro kit (Stratagene). The isolated total RNA was DNaseI-digested to remove genomic contamination and first strand cDNA was synthesized using oligo(dT) primers and Omniscript reverse transcriptase (Qiagen). The real time PCR reactions were carried out in an ABI Prism 7000 sequence detector (Applied Biosystems) using the Gold 2×SYBR green reaction mix (Eurogentec). Each assay contained a duplicate and a minus RT control. The efficiency for each real time PCR run was determined according to [28]. The expression of the gene of interest was subsequently normalized to the expression of the housekeeping genes *RNA Polymerase II* or *Ubiquitin C* [29]. Normalization of expression data was computed by the qGENE tool [30]. All real time PCR amplicons were set across an exon-exon boundary. Primers used in real time PCR reaction:

RPII F: TGCGCACCACGTCCAATGATA;

RPII R: AGGAGCGCCAAATGCCGATAA;

UBC F: AGGTCAAACAGGAAGACAGACGTA;

UBC R: TCACACCCAAGAACAAGCACA;

18s F: GCCCGAGCCGCCTGGATACC;

18s R: TCACCTCTAGCGGCGCAATACGAA;

*λ5* F: GAGACAACCCAACCCTCCAA;

*λ5* R: TGAGGCATCCACTGGTCAGA;

cy GSN F: CCGTCGCTGTCGCCGTCA;

cy GSN R: GCACCAGGTCAAACTTCTCCACAC;

Aio F: TCCTGGACAGATTAGCAAGCAA;

Aio R: GGGATTGTAGTTGCCATCGAA;

mOBF-1 F: CACGCCCAGTCACATTAAAGAA;

mOBF-1 R: TGTGGATTTTTGCCAGAGCAT;

### RNA and RT-PCR

Total RNA was isolated from 1×10^5^ sorted cells and cDNA synthesized as above. Immunoglobulin *κ* cDNA was amplified with 30 cycles at 62°C annealing temperature using the following primers:

Vκ deg F: GGCTGCAGSTTCAGTGGCAGTGGRTCWGGRAC;

Cκ R: GTCCTGATCAGTCCAACTGTTCAG;

### Cell cycle analysis

Bone marrow pre-BI, large and small pre-BII and immature B cells were isolated by cell sorting and fixed in 1% paraformaldehyde at 4°C overnight. After permeabilization with 70% ethanol at −20°C the cells were stained with 50 µg/ml Propidium Iodide (Sigma, P 4170) in PBS and treated with 0.1 mg/ml RNase A. The cells were analyzed FACScalibur and the recorded data analyzed with the ModFit software (BD Immunocytometry Systems).

### Genomic rearrangement of the *κ* locus

B220^+^ CD25^+^ IgM^−^ pre-BII cells were isolated from five age-matched animals of each genotype by cell sorting. Genomic DNA was isolated and equal amount of DNA was used in serial dilutions to detect *Vκ-Jκ* rearrangements. For this, a universal *Vκ* primer [31] was used in combination with a specific *Jκ2* or *Jκ5*. The *Vκ-Jκ* DNA was amplified in a multiplex PCR for 35 cycles at 60°C annealing temperature using the specific *Vκ1,2* primer together with *Jκ1* and *Jκ2,3* primers.

Vκ universal F: GGCTGCAGSTTCAGTGGCAGTGGRTCWGGRAC;

Jκ2 R: TTTCCAGCTTGGTCCCCCCTCCGAA;

Jκ5 R: TGCCACGTCAACTGATAATGAGCCCTCTC;

Vκ1,2 F: AGAAGCTTGTGACTCAGGAATCTGCA;

Jκ1 R: CAGGATCCTAGGACAGTCAGTTTGGT;

Jκ2,3 R: CAGGATCCTAGGACAGTGACCTTG;

### Affymetrix GeneChip analysis

Pre-BII cells (B220^+^ CD25^+^) were isolated from pooled bone marrow of eight age-matched mice from each genotype. From each genotype cells from two independent pools were purified. Subsequently, total RNA was extracted as above and further purified with the RNAeasy mini columns (Qiagen). Starting with 4 mg of total RNA, biotinylated cRNA probes were generated using the SuperScript Choice cDNA sythesis kit from Stratagene and the Affymetrix IVT kit. The labeled cRNA probes were hybridized with the MOE430a Affymetrix GeneChips™ and signals were detected according to manufacturer's instructions. The GeneChips data analysis was performed using the Affymetrix Microarray suite 5 and the GeneSpring 5 (Agilent Technologies, Palo Alto, CA) analysis package. Only target intensities between 100 and 1000 in at least one genotype were used in the analysis. The microarray data has been deposited in Gene Expression Omnibus (GEO) system. The accession number is: GSE12356.

### Chromatin immunoprecipitation assay

Crosslinking with formaldehyde and chromatin isolation from A20J B cell line was performed as described [32]. Chromatin was sonicated to an average fragment size from 200 to 600 bp followed by 1∶4-dilution before immunoprecipitation with 5 µg of anti-OBF-1 (Santa Cruz) antibody. As a negative control, the chromatin was immunoprecipitated with goat IgG isotype control (Sigma). After crosslinking reversal, the immunoprecipitated DNA was analyzed by real time PCR using the relative standard curve method for normalization. The following primers were used in the qPCR reactions:

ChIP PBS1 F: GGATAGACTTTGCATGTTTTTGAT;

ChIP PBS1 R: GGGGGATATTACCTGCTCTTT;

ChIP PBS2 F: CCTGGACTTGCCCATTTTTA;

ChIP PBS2 R: ATGATGGAATGCAGGTGACA;

ChIP PBS3 F: CTTTCTGCCACCACCTC TC;

ChIP PBS3 R: CTCCATGACCCTCAAATACCA;

ChIP Interg F: CAGCATTCCAGGAGGTTAGC;

ChIP Interg R: GTGCCTCATGTGCAGTCAGT;

### 3D DNA FISH

3D DNA FISH was carried out as described previously [33]. A probe specific for *γ-satellite* DNA labeled with dUTP-Cy5 and a BAC clone (RP24–166A3, BAC PAC Resources) for *VpreB1/λ5*, labeled with dUTP-Cy3 were used [34]. Cells were analyzed by confocal microscopy on a Leica SP2 AOBS (Acousta Optical Beam Splitter). Optical sections were collected with sections separated by a distance of 0.3 mm. Only cells with two *λ5* signals (non-replicating) were evaluated.

### Retroviral vectors and infections

The expression vector pBabe/puro/*Bcl2* was provided by Dr. S. Nutt (The Walter and Eliza Hall Institute, Australia). The following primers were used for amplification:

VpreB1 XhoI F: AATTATCTCGAGGTCAGGGCCCAGGAGCAG

VpreB1 EcoRI R: AATCATGAATTCAGACTAGCCGAGCAAAGCAAACT

IRES BglII F: CTTGAGATCTGGGATCCGCC

IRES XhoI R: AATATCCTCGAGATTATCATCGTG

*λ5* clone 2 F: CCTGGGGTTTGGCTACAC

*λ5* clone 2 R: GAGGCTTCAGGGAGGATT

The *VpreB1* cDNA was amplified by PCR and inserted between the XhoI and EcoRI sites of the retroviral vector pMSCV/pgk/*EGFP*. Subsequently, a 597 bp fragment containing an internal ribosomal entry site was amplified by PCR from the retroviral vector pMSCV/IRES/*hCD2t* and inserted into the BglII and XhoI sites of pMSCV/*VpreB1*/pgk/*EGFP*. Finally, the *λ5* cDNA was amplified by PCR and inserted upstream of the IRES sequence into the HincII site of pMSCV/IRES/*VpreB1*/pgk/*EGFP*, leading to pMSCV/*λ5*/IRES/*VpreB1*/pgk/*EGFP*.

Phoenix-Eco packaging cells were transfected with pBabe/puro/*Bcl2*, pMSCV/pgk/*EGFP*, or pMSCV/*λ5*/IRES/*VpreB1*/pgk/*EGFP* retroviral vectors by using calcium phosphate (Promega, Madison, USA) according to the manufacturer's protocol. Retroviral supernatants from of Phoenix-Eco packaging cells cultured at 37°C were collected after 48 h. Next, the target cells were centrifuged for 90 min at 1800 rpm at room temperature in a retroviral supernatant. Cells were incubated for 90 min at 37°C and submitted to another round of spin infection with fresh retroviral supernatant.

### *In vitro* maturation of pro/pre-B cells

5′ Flourouracil treated bone marrow cells from *C57BL/6* mice were transduced and cultured as previously published [35]. In short: 5′ FU treated cells were transduced with pBabe/puro/*Bcl2* and selected for Bcl2 expression by puromycin. Subsequently the cells were cultured on OP9 stromal cells with IL-7 and Flt3 to differentiate into pro/pre-B cells. These pro/pre-B cells were transduced with either pMSCV/*λ5*/IRES/*VpreB1*/pgk/*EGFP* or the empty *EGFP* retroviral expression constructs. The transduced cells were cultured without IL-7 on OP9 stromal cells for 5 or 7 days to induce further B cell maturation. The maturation state of the transduced GFP^+^ cells was determined by detection of surface κ light chain expression by flow cytometery.

## Results

### Transition from small pre-BII cells to immature B cells is impaired in Aiolos/OBF-1 double mutant mice

**Figure 1:**
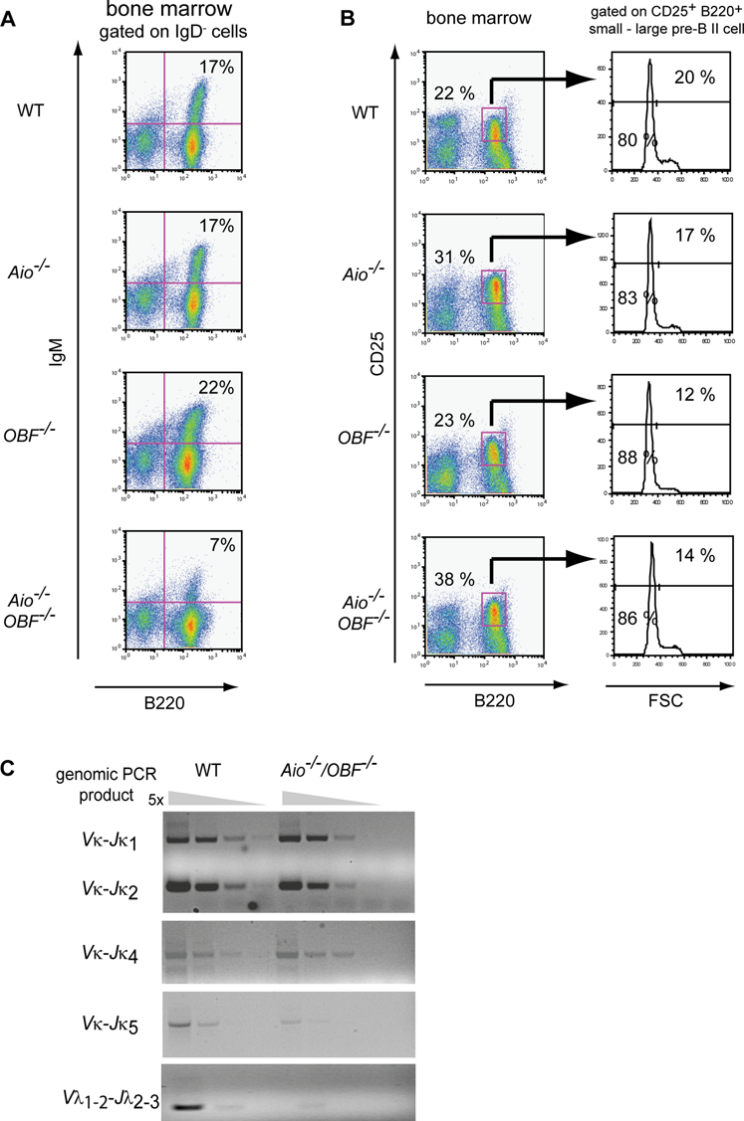
The transition from small pre-BII to immature B cells is impaired in *Aio^−/−^/OBF-1^−/−^* mice. Flow cytometry analysis of bone marrow cells from 6–10 weeks old control wild type, single- and double-deficient mice. (A) Analysis of B220^+^ IgM^+^ IgD^−^ immature B cells in the bone marrow. Cells were stained with anti-B220-APC, anti-IgM-FITC, anti-IgD-Biotin followed by Streptavidin-PE. Only IgD^−^ cells are displayed and the percentages indicated are relative to the IgD^−^ cells. The percentage of IgD^−^ cells from total cells are as follows: WT, 89%; *Aiolos^−/−^*, 88%; *OBF-1^−/−^*, 92%; *Aio^−/−^/OBF-1^−/−^*, 95%. (B) In the left part detection of B220^+^ CD25^+^ pre-BII B cells. In the right part, pre-BII (B220^+^CD25^+^) cells were gated and their FSC analyzed. Percentages indicate the proportion of small or large cells. Data are presented from one representative experiment, out of three. (C) Pre-BII cells (B220^+^ CD25^+^ IgM^−^) were FACS sorted from wild type and Aiolos/OBF-1 double-deficient mice by flow cytometry. Serial dilutions of the genomic DNA from the sorted cells were analyzed by PCR using primers detecting the indicated *κ* and *λ* light chain rearrangements. The relative amount of DNA used in the reaction was adjusted according to a real time PCR for the *18S* gene (not shown).

The loss of either Aiolos or OBF-1 results in a reduction of immature splenic B cells and impaired formation of MZ B cells [10], [15], [22]. We have shown previously that the number of mature resting B cells is only marginally altered in the single-mutant mice, but is reduced several-fold in *Aiolos^−/−^/OBF-1^−/−^* mice [21]. To better understand the molecular basis of this phenomenon, we have examined here early B cell development in mice of the different genotypes. In agreement with our previous studies [21], we found that immature B cells (B220^+^ IgM^+^ IgD^−^) are severely reduced in Aiolos/OBF-1 double - but not single-deficient or wild type mice (Figure 1A). As previously reported [27], Aiolos single-deficient mice showed an increase in the proportion of pre-BII (B220^+^ CD25^+^ IgM^−^) cells. Moreover, the double-deficient mice exhibited a further small increase in the proportion of pre-BII cells, although OBF-1 single-deficient mice showed no change in this cell population (Figure 1B). To distinguish between the dividing and non-dividing, but light chain rearranging cells, pre-BII cells can be fractionated on the basis of their size (FSC). Independent of genotype, about 80 to 90% of the pre-BII cell population represented small non-dividing cells (Figure 1B), which was also confirmed by DNA content analysis from sorted pre-BII cell populations (data not shown).

Small pre-BII cells were used to determine the DNA rearrangement status of the *Ig κ*, *λ1*, and *λ2* light chain genes. We observed no difference in the rearrangement of *Vκs* to *Jκ1*, *Jκ2* or *Jκ4* between wild type and double-mutant pre-BII cells (Figure 1C). However, *Aiolos^−/−^/OBF-1^−/−^* pre-BII cells showed a five-fold reduction in *Jκ5* rearrangement compared to pre-BII cells from control wild type littermates. Furthermore, the rearrangement of the *λ1* and *λ2* genes was also reduced at least fivefold in the double-mutant pre-BII cells. The reduction in *Vκ-Jκ5* and *λ* light chain gene rearrangements in Aiolos/OBF-1 deficient mice suggests that the recombination process is terminated prematurely during the small pre-BII cell stage. In spite of this, *κ* light chain rearrangement occurs in double-mutant mice and intracellular expression of *κ* light chains was detected in the pre-BII cell population of these mice (data not shown). The increase in small pre-BII cells concomitant with the reduction in immature cells indicate that in the combined absence of Aiolos and OBF-1 B cell development is impaired at the transition from the small pre-BII to immature B cell stage.

### Expression of Aiolos and OBF-1 during early B cell development and influence of these factors on the gene expression profiles of pre-BII cells

**Figure 2:**
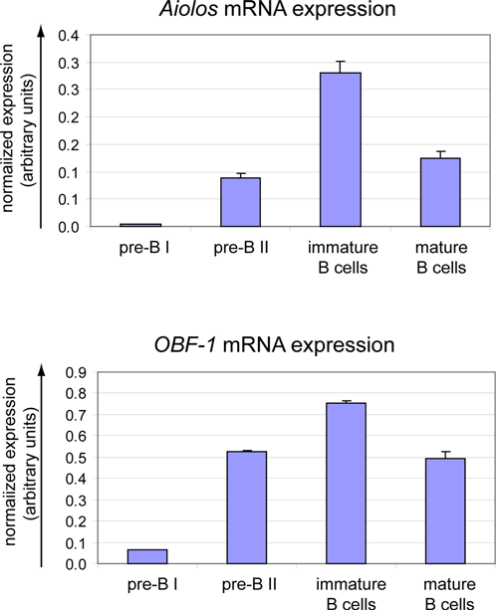
Expression pattern of *Aiolos* and *OBF-1* in the bone marrow. The indicated B cell fractions were sorted by FACS and expression of *Aiolos* and *OBF-1* was determined by real time RT-PCR and normalized to the expression of *Ubiquitin C*. Similar results were obtained when using *RPII* for normalization. The histograms represent the mean±SE based on the analysis of at least three independent samples per stage.

**Figure 3:**
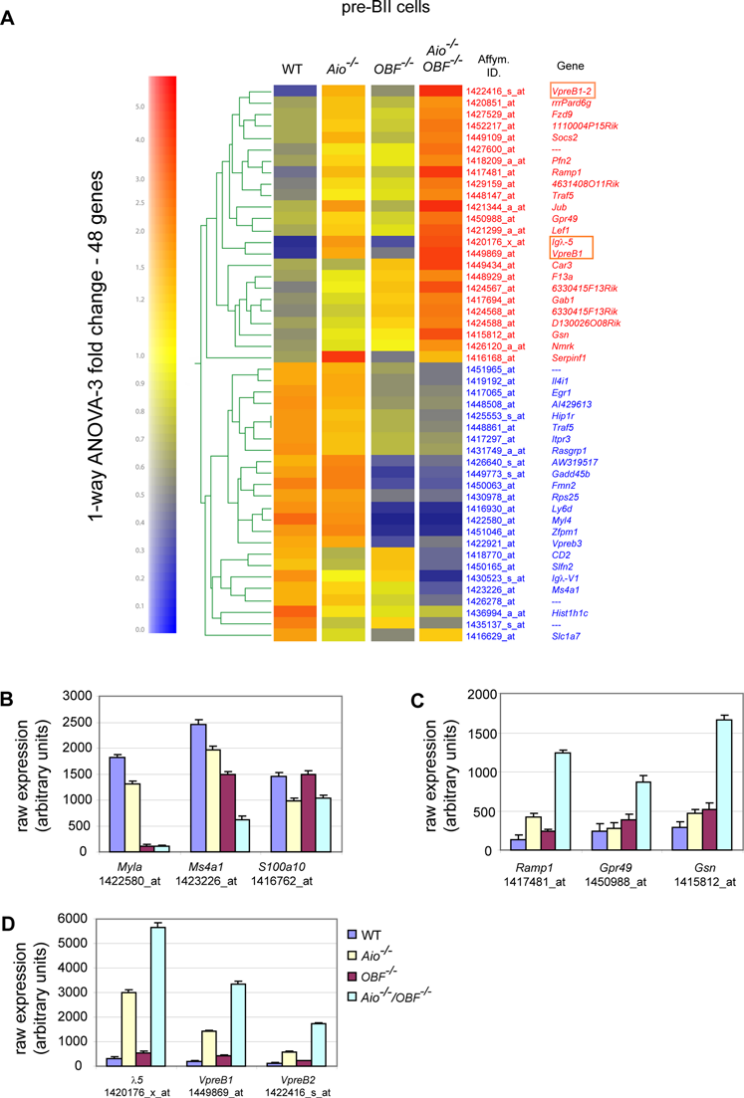
Identification of genes regulated by Aiolos and OBF-1 in pre-BII cells. Gene expression profiles in B220^+^CD25^+^ pre-BII cells were determined by MOE430a Affymetrix GeneChip; for each genotype two RNA samples were prepared from independent pools of mice and microarray analysis was done in duplicate. (A) 48 genes showed a 3 fold expression changes in single- or double-mutant pre-BII cells compared to pre-BII cells from wild type mice (p-value cutoff: 0.05). The genes are grouped according to their expression profile in pre-BII cells from all genotypes. Low mRNA expression, blue; high mRNA expression, red. (B) Expression of genes that have been reported previously to be dependent on OBF-1 expression: *Myla*, *Ms4a1*, *S100a10*. (C) Expression of genes that show a strong expression increase specifically in *Aio^−/−^/OBF-1^−/−^* pre-B cells: *Ramp1*, *Gpr49*, *Gelsolin* (*Gsn*). (D) Expression of the surrogate light chain genes: *λ5*, *VpreB1* and *VpreB2*. Figures show raw Affymetrix expression score after array normalization.

We next investigated by quantitative RT-PCR the expression pattern of OBF-1 and Aiolos during B cell development. Both genes showed a similar pattern of expression: low in pre-BI, intermediate in pre-BII, high in immature B and intermediate in mature B cells (Figure 2), thus supporting a role for these factors in early B cell development. In order to determine the role of OBF-1 and Aiolos in pre-BII cells, the mRNA expression profile in pre-BII cells from wild type, Aiolos or OBF-1 single-mutant and double-mutant mice were measured, using the MOE430A Affymetrix GeneChip (Figure 3A). Previous mRNA expression analysis of OBF-1 deficient B cells have indicated a role for this factor in regulating several genes, including *Lck*, *Kcnn4*, *cdc37*, *Myla*, *Ms4a11* or *S100a10* [36], [37], some of which were confirmed by our present analysis (see Figure 3B). In comparing the transcriptomes in cells of the four genotypes we found that the majority of genes with a reduced mRNA expression in the double-mutant pre-BII showed a similarly diminished expression in *OBF-1^−/−^* pre-BII cells. Most of the genes that were upregulated in the double-mutant pre-BII, were also partially upregulated in pre-BII cells from either single-mutant mice. We found that the expression of some genes, like *RAMP1* (calcitonin receptor-activity modifying protein 1), *Gpr49* (a G-protein-coupled receptor), or *Gsn* (Gelsolin, an actin binding and severing protein) was found to be synergistically upregulated in the pre-BII cells from double-deficient mice (Figure 3C). The mRNA expression of the major players in immunoglobulin gene rearrangement (*RAG1-2*, *dntt*, *Ku70*, *polm*) was not altered by the absence of Aiolos, OBF-1 or both factors (data not shown). Strikingly, loss of Aiolos and OBF-1 altered the expression of the genes encoding the SLC, *λ5*, *VpreB1* and *VpreB2* (Figure 3D). The role of Ikaros and Aiolos in regulating expression of *λ5* was recently demonstrated [38], [39]. This regulatory role was confirmed by our observations of a 10-fold increase of *λ5* mRNA expression levels in Aiolos deficient pre-BII cells. Furthermore, the mRNA expression levels of *VpreB1* and *VpreB2* were increased up to 7-fold in Aiolos deficient cells, thus demonstrating a regulatory role of Aiolos for all members of the surrogate light chain. Although *λ5* and *VpreB1,2* mRNA levels were only moderately elevated (2-fold) in OBF-1 deficient pre-BII cells, expression of these genes was increased up to 25-fold in pre-BII cells of the double-mutant mice (Figure 3D) indicating that *λ5*, *VpreB1* and *VpreB2* depend on both Aiolos and OBF-1 for their correct expression.

### Pre-BII cells lacking Aiolos and OBF-1 fail to downregulate the components of the surrogate light chain

**Figure 4:**
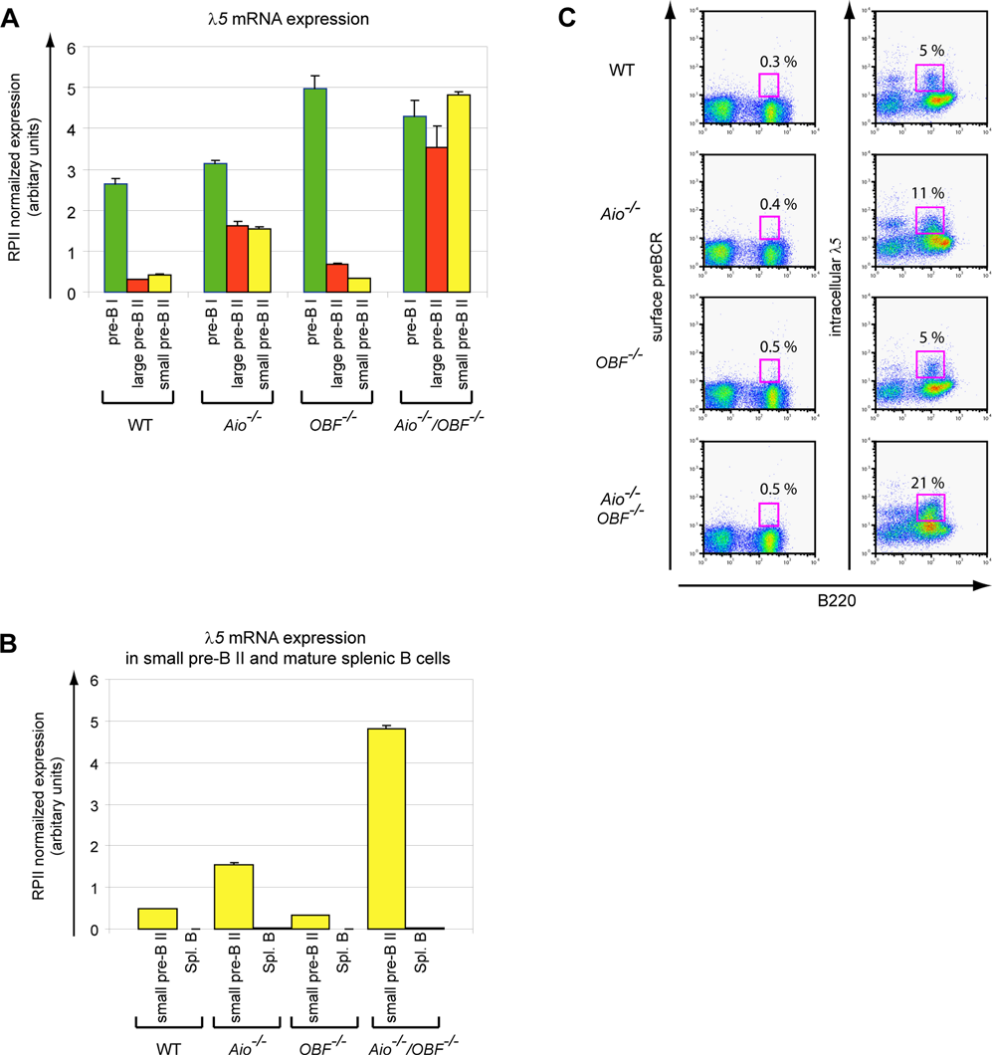
Silencing of *λ5* expression at the pre-BII cell stage is impaired in absence of Aiolos and OBF-1. (A) *λ5* expression in pre-BI and pre-BII cells of the different genotypes. Pre-BI cells (B220^+^ckit^+^IgM^−^), large or small pre-BII cells (B220^+^CD25^+^IgM^−^, discriminated on the basis of their FSC profile) were sorted from wild type, single- and double-deficient mice and *λ5* expression was analyzed by real time RT-PCR. The histograms represent the mean±SE based on the analysis of at least three independent samples per genotype/stage. (B) Downregulation of *λ5* expression in *Aiolos^−/−^/OBF-1^−/−^* splenic mature B cells. Small pre-BII (B220^+^CD25^+^IgM^−^) and splenic mature B (B220^+^IgM^low^IgD^high^) cells of wild type, single- and double-deficient mice were sorted and the expression analysis was done by real time RT-PCR, as above. The histograms represent the mean±SE based on the analysis of two to three independent samples per genotype/stage. (C) Aiolos/OBF-1 double-deficient pre-BII cells fail to downregulate *λ5* expression, but have a normal pre-BCR expression at the cell surface. Bone marrow cells from wild type, single- and double-mutant mice were stained for B220 together with surface pre-BCR (left panels) or intracellular surrogate light chain (λ5, right panels). Representative stainings are presented.

The combined expression data from pooled large and small pre-BII cells demonstrated strikingly elevated levels of *VpreB1*, *2* and *λ5* mRNA in Aiolos single- and Aiolos/OBF-1 double-mutant mice. To determine whether this was the result of a net increase in transcription or a failure to properly silence these loci during development, *λ5* mRNA expression was determined by quantitative RT-PCR in pre-BI cells, as well as in large or small pre-BII cells (Figure 4A). In wild type animals the *λ5* gene was highly expressed at the pre-BI cell stage and subsequently downregulated more than ten-fold in large and small pre-BII cells, in agreement with previous results [6], [40]. Aiolos single-mutant mice showed normal *λ5* mRNA levels in pre-BI cells, indicating that at this stage Aiolos does not influence expression of the *λ5* gene. In contrast, in large or small pre-BII cells from *Aiolos^−/−^* mice, *λ5* mRNA levels were reduced only two-fold compared to the pre-BI stage. The absence of OBF-1 led to a striking two-fold increase in *λ5* expression in pre-BI cells, but the downregulation in large and small pre-BII cells was almost normal. Remarkably, the absence of both Aiolos and OBF-1 resulted in elevated *λ5* mRNA levels in pre-BI cells, which were sustained in both large and small pre-BII cells (Figure 4A). In order to determine whether *Aiolos^−/−^/OBF-1^−/−^* mice are able to silence the expression of the *λ5* gene at all, the expression of *λ5* mRNA was determined in splenic mature resting B cells. As shown in Figure 4B, *λ5* mRNA was not detected in splenic B cells, irrespective of genotype (See also Supplemental Figure S1A). Thus, an additional silencing mechanism exists, which is independent of Aiolos and OBF-1. In contrast to this, the *Gelsolin* gene, which also showed increased expression levels in the bone marrow of mutant mice, maintained elevated levels in splenic B cells (Supplemental Figure S1A–B). This indicates that in *Aiolos^−/−^/OBF-1^−/−^* mice, the few B cells that migrate to the spleen and develop into mature B cells, successfully silence the *λ5* locus, but are still deregulated in the mRNA expression of other Aiolos/OBF-1 dependent genes.

In wild type pre-BII cells the pre-BCR has a high turnover and most is retained in the endoplasmatic reticulum, resulting in low expression at the cell surface [41], [42]. However, mice with impaired pre-BCR signaling, such as SLP65 or Irf4/Irf8 mutant mice, show increased surface expression levels of the pre-BCR due to impaired receptor internalization [43]. In order to test for a change in surface pre-BCR or intracellular λ5 expression, bone marrow cells from wild type and mutant mice were stained with specific antibodies against the pre-BCR and the λ5 chain. By flow cytometry analysis the single-and double-mutant mice showed no significant increase in pre-BCR expression at the surface of bone marrow B-lineage cells (Figure 4C). In contrast, the proportion of B-lineage cells expressing intracellular λ5 was two-or four-fold increased in Aiolos and double-mutant mice, respectively, whereas λ5 protein levels determined by mean fluorescence levels in flow cytometry were only marginally elevated (Figure 4C). Thus, the increase in small, but not large, pre-BII cells in Aiolos and double deficient mice indicates that the observed increase in *λ5* mRNA and protein levels is not due to an accumulation of early B cells that normally express *λ5* at higher levels, but rather is caused by inappropriately high expression of the *λ5* locus in small pre-BII cells. Furthermore, although *λ5* mRNA and protein levels are higher in pre-BII B cells from Aiolos and double-mutant mice, the mechanism of pre-BCR internalization is not impaired in the pre-BII cells from these mice.

### Direct regulation of *λ5* by Aiolos and OBF-1

**Figure 5:**
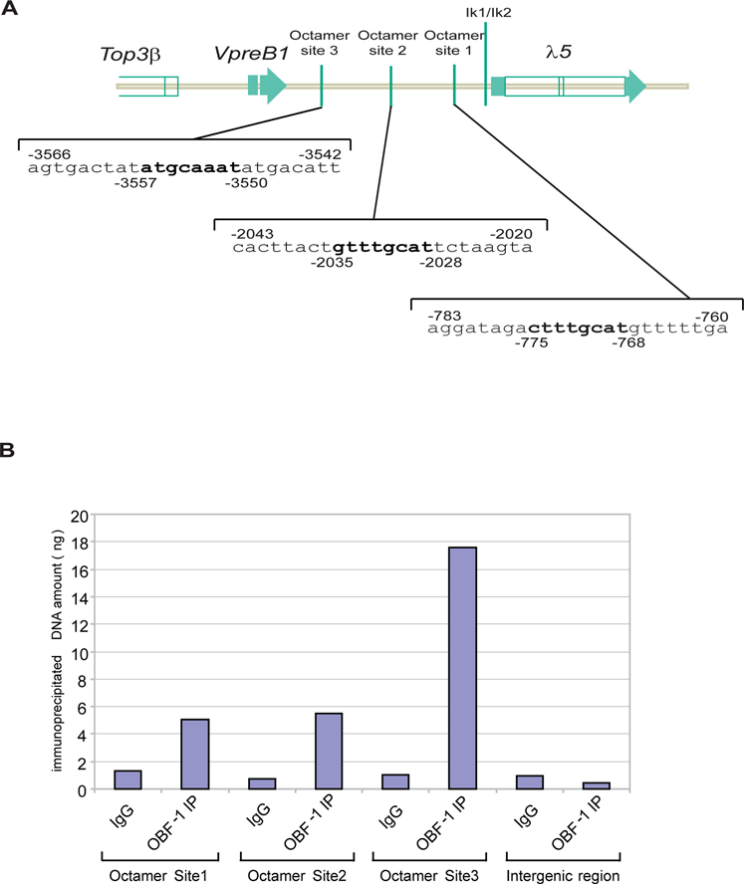
OBF-1 binds to the *λ5* promoter region *in vivo*. (A) Organization of the *λ5* locus and partial sequence of the *λ5* promoter region. Three putative octamer sites were identified *in silico*. (B) OBF-1 binds to the *λ5* regulatory region. Binding of OBF-1 to three putative octamer sites (−769 to −776), (−2029 to −2036) and (−3557 to −3550) was tested by ChIP analysis. As negative controls, ChIP assays were performed with control IgGs, and control amplifications were done with a fragment of an intergenic region from chromosome 8 lacking any octamer site. Immunoprecipitated DNA was quantified by real time PCR. One representative experiment is presented.

In mice the *λ5* and *VpreB* genes are located on chromosome 16; *VpreB1* and *λ5* are separated by 4 kb and *VpreB2* is located 1 Mb downstream of the *VpreB1-λ5* locus. This region also contains a gene encoding the ubiquitously expressed *TopoisomeraseIIIβ* (*Top3β*) which is located only 1.5 kb upstream from *VpreB1* (Figure 5A). Expression of the *λ5* and *VpreB* genes is initiated by and dependent on the basic helix–loop–helix proteins E12 and E47 and on the zinc finger/helix-loop-helix early B-cell factor (EBF) [44]. Although there are indications for a direct interaction of Aiolos/Ikaros with the *λ5* promoter/enhancer region [38], [45], no direct interaction of OBF-1 with the *λ5* locus has been documented so far. Bioinformatics analysis using PROMO [46] revealed three putative octamer binding sites (Octamer 1: CTTTGCAT (−768 to −775), Octamer 2: GTTTGCAT (−2028 to −2035) and Octamer 3: ATGCAAAT (−3557 to −3550; consensus –ATTTGCAT-) in the *λ5* 5′ regulatory region [47] (Figure 5A).

To determine whether OBF-1 is bound to these sites *in vivo*, the B lymphoma line A20J was used for chromatin immunoprecipitation (ChIP) assays. Binding of OBF-1 was detected by ChIP using an anti-OBF-1 antibody and formaldehyde-crosslinked chromatin from A20J cells. Detection of the immunoprecipitated DNA was done with a quantitative PCR assay. As shown in Figure 5B, the two proximal octamer sites (sites 1 and 2) were both enriched 5 to 6 fold in comparison to a ChIP done with control IgG. In the same assay, the distal octamer site was even enriched about 18 fold. Thus, all three identified octamer sites in the *λ5* regulatory region bind OBF-1 in B cells. These findings demonstrate that OBF-1 can bind to the regulatory region of the *λ5* locus in B cells and therefore may directly regulate *λ5* transcription.

### The developmental repositioning of the *λ5* locus is dependent on Aiolos and OBF-1

Recent studies by Parker et al. [34] have shown that the *VpreB/λ5* genes are silenced in an asynchronous manner. Using RNA and DNA FISH analysis, it was demonstrated that the *VpreB/λ5* genes are bi-allelically transcribed in pre-BI (CD19^+^ c-kit^+^), mono-allelically in large pre-BII (B220^+^ CD25^+^ IgM^−^) and are subsequently completely repressed in small pre-BII cells (B220^+^ CD25^+^ IgM^−^). This correlates with a sequential recruitment of the *VpreB/λ5* alleles to centromeric DNA and translocation to the nuclear periphery during early B cell development. Several earlier studies have shown that recruitement of genes to heterochromatin and repositioning to the nuclear periphery is a prerequisite for their transcriptional silencing [48]–[50].

**Figure 6:**
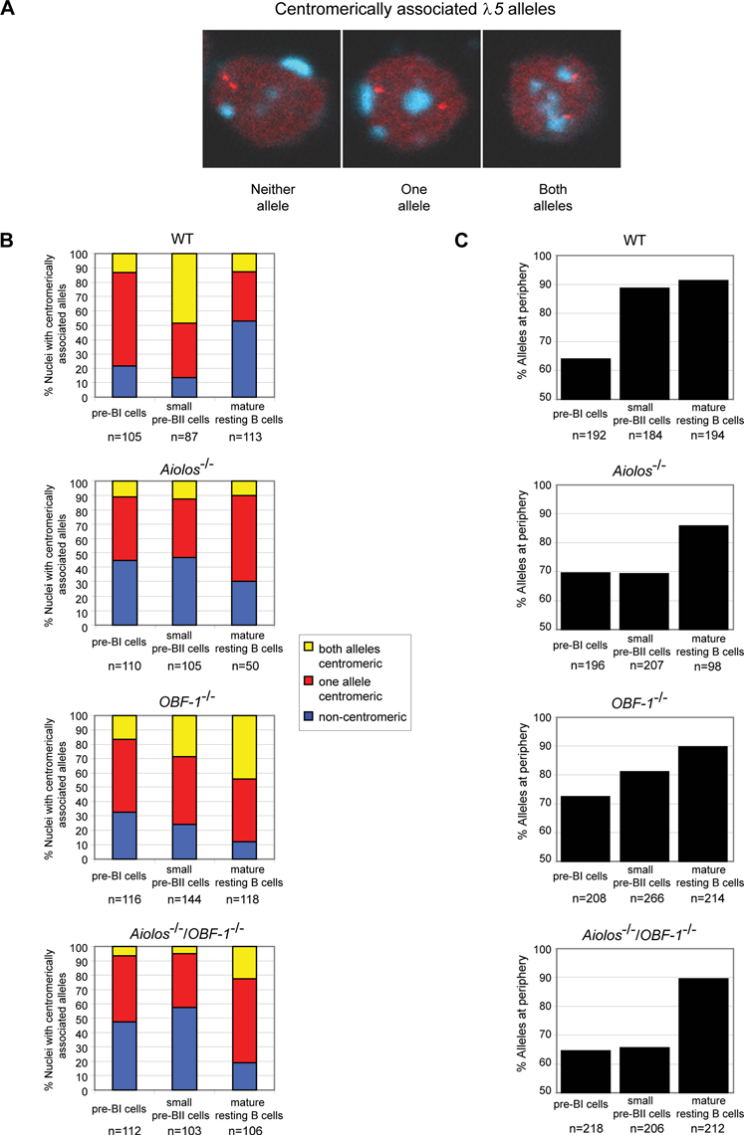
Developmentally regulated nuclear repositioning of the *λ5* locus is impaired in B lymphocytes lacking Aiolos or OBF-1. (A) Representative pictures of the DNA FISH analysis. Confocal sections of nuclei after DNA FISH are shown, combining the *λ5* probe (red staining) with a probe detecting *γ-satellite* DNA (blue staining). The pictures show association of zero, one or both *λ5* alleles with *γ-satellite* DNA. (B) Localization of the *λ5* locus determined by DNA FISH analysis of bone marrow pre-BI (B220^+^ c-kit^+^ IgM^−^), small pre-BII (B220^+^ CD25^+^ IgM^−^), and splenic resting mature B cells (CD4^−^ CD43^−^ Ter^−^ 119^−^). Percentages of nuclei with one (red bar), both (yellow bar) or neither (blue bar) *λ5* allele associated with *γ-satellite* DNA in the indicated populations of wild type, single-and double-deficient mice. (C) Percentages of alleles located at the nuclear periphery in the indicated cell populations.

In order to determine the impact of Aiolos and OBF-1 on this process, 3D DNA FISH was carried out on sorted pre-BI, small pre-BII as well as splenic mature resting B cells from wild type and mutant mice. A *γ-satellite* probe was used to detect centromeric clusters in conjunction with a *VpreB/λ5* locus probe (Figure 6A). In wild-type pre-BI cells, the majority (65%) of cells has only one *VpreB1*/*λ5* allele colocalizing with centromeric DNA and only a small proportion (13%) of the cells show both alleles positioned at the centromeric heterochromatin (Figure 6B). In the course of B cell development, a shift can be observed, as the *VpreB1*/*λ5* alleles become progressively recruited to the centromeric DNA at the small pre-BII cell stage: at this stage a much greater proportion (48%) of the cells have both alleles associated with centromeric DNA and a minority of the cells (38%) have only one allele centromeric. Finally, in the majority of mature splenic B cells the *VpreB1/λ5* alleles are no longer centromerically-associated. In addition, the recruitment to centromeric DNA is accompanied by translocation of the alleles to the nuclear periphery: while ca. 60% of *VpreB/λ5* alleles are peripheral in wild type pre-BI cells, in small pre-BII or mature splenic B cells about 90% of the alleles are at the nuclear periphery (Figure 6C).

In contrast to the wild-type, *Aiolos* deficient cells show a delayed recruitment of alleles to the centromeric DNA at the small pre-BII stage: a much smaller proportion of these cells have both *VpreB1/λ5* alleles centromerically associated (12%, compared to 48% for the wild type). Conversely, a much larger proportion of the cells (47%) have the *VpreB1/λ5* alleles not associated with centromeric DNA (Fig. 6B). Furthermore, the proportion of *Aiolos^−/−^ VpreB1/λ5* alleles relocalizing at the periphery does not increase in small pre-BII cells and remains at ca. 70% (Fig. 6C). Yet, despite this delayed recruitment at the small pre-BII stage, the majority (85%) of the *VpreB1/λ5* alleles have repositioned to the nuclear periphery in mature splenic *Aiolos^−/−^* B cells, as in wild type cells. Therefore, delayed recruitment is observed only in the small pre-BII population.

Similarly to the loss of Aiolos, the lack of OBF-1 leads to a delayed centromeric recruitment and repositioning to the nuclear periphery of the *VpreB1/λ5* alleles, albeit less severe (Figure 6B): compared to *Aiolos^−/−^*, a greater proportion of the *OBF-1^−/−^* pre-BII cells have both *VpreB1/λ5* alleles located at the centromere (28%, vs. 12% for *Aiolos^−/−^* cells). Furthermore, the proportion of cells having both alleles located away from the centromeres is smaller than in the case of the Aiolos mutation (24%, vs. 47% for *Aiolos^−/−^* cells). Likewise, the level of peripheral localization is greater than that observed in *Aiolos^−/−^* small pre-BII cells, but less than what is seen in wild type cells.

Finally, the analysis of cells from the Aiolos/OBF-1 double-mutant mice revealed that in the absence of both factors the observed phenotype of delayed recruitment and relocalisation of alleles is further enhanced: a much smaller proportion (only 5%) of the small pre-BII cells have both *VpreB1/λ5* alleles associated with centromeric repeats and a much larger proportion of cells have the *VpreB1/λ5* alleles entirely non-centromeric (57%). Likewise, peripheral localization is further reduced in small pre-BII cells (Figure 6C). Together, these results indicate that the relocalization of *VpreB1/λ5* alleles to centromeric heterochromatin and their repositioning to the nuclear periphery depends primarily on Aiolos but that OBF-1 also contributes to the process.

### High VpreB1 and λ5 expression interferes with B cell maturation *in vitro*

**Figure 7:**
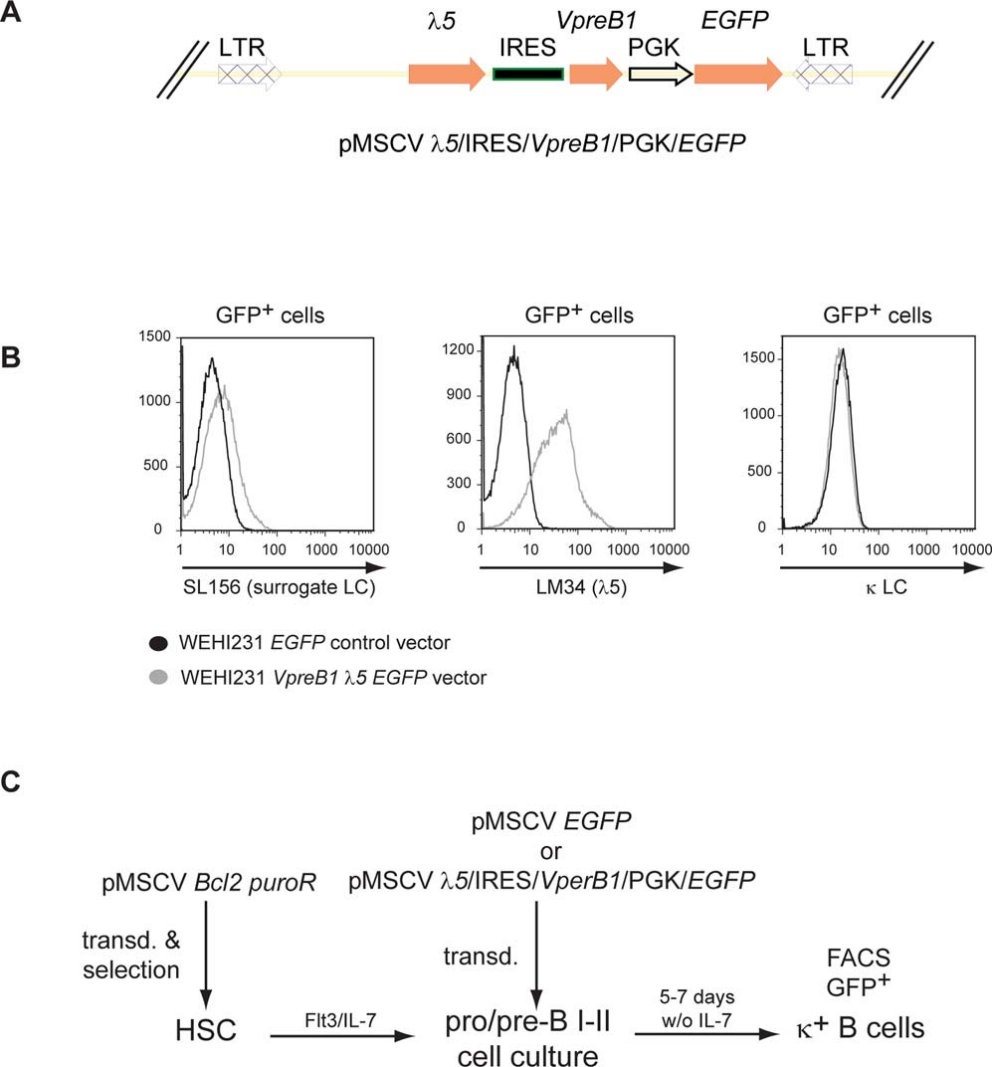
Assessing the role of VpreB1 and λ5 expression for pro B cells maturation *in vitro*. (A) In the upper part a schematic of the retroviral construct used to transduce pro/pre-B cells is presented. The control construct is identical, but only contains EGFP. (B) WEHI231 lymphoma B cells were transduced with either *VpreB1-IRES-λ5-EGFP* or an *EGFP* retroviral expression construct. Expression of VpreB1 and *λ*5 was determined by staining for surface expression of the pre-BCR (SL156) and *λ*5 (LM34). Possible displacement of κLC on transduced WEHI231 cells was determined by staining for surface κLC. Representative stainings are presented. (C) Schematic of the transduction experiment with bone marrow cells. Hematopoietic stem cells (HSC) were first transduced with a Bcl-2 expressing retrovirus to enhance their survival and were differentiated into pro/preB cultures in the presence of Flt3 and IL-7. Subsequently, these cultures were transduced with the indicated retroviruses and differentiated *in vitro* by withdrawing IL-7.

The expression of members of the surrogate light chain VpreB1, 2 and λ5 is tightly controlled at the transition from pre-BI, large and small pre-BII, to immature B cells, suggesting that this downregulation is essential. In order to test whether high levels of the surrogate light chain could interfere with the maturation of early B cell stages, we set out to overexpress λ5 and VpreB1 in early B cells, using a *λ5-IRES-VpreB1-EGFP* retroviral expression vector (Figure 7A). First, expression of VpreB1 and λ5 from the *λ5-IRES-VpreB1-EGFP* retroviral vector was demonstrated by transducing the immature B cell lymphoma WEHI231 and staining for surface expression of total pre-BCR or λ5 (Figure 7B). Although the SL and endogenous kappa light chain (κLC) can compete for intracellular μHC, no reduction of surface κLC was detected in *λ5-IRES-VpreB1-EGFP* transduced WEHI231 cells, indicating an excess of endogenous intracellular μHC (Figure 7B). Next, IL-7 dependent pro/pre-B cell cultures were transduced with a construct expressing *λ5-IRES-VpreB1-EGFP*, or with a control construct expressing only EGFP (see experimental scheme in Figure 7C). Removal of IL-7 promotes maturation of the cultured pro/pre-B cells into immature B cells which express surface IgM [51] and κLC on their cell surface. After 5 to 7 days culture in the absence of IL-7, *λ5-IRES-VpreB1-EGFP* transduced cells produced only ∼50% of surface κLC^+^ cells compared to control transduced cells expressing only EGFP (Table 1). Hence, overexpression of the surrogate light chain interferes with the maturation of pro/pre-B cells to more mature κLC^+^ expressing B cells *in vitro*.

**Table 1:** Expression of VpreB1 and λ5 in pro-B cells impairs their maturation.

		CD19^+^ GFP^+^ κ^−^	CD19^+^ GFP^+^ κ^+^
**Experiment 1** 7 days w/o IL-7	Control vector	76.3%	23.7%
	*VpreB1 λ5* vector	85.3%	14.7%
**Experiment 2** 5 days w/o IL-7	Control vector	83.3%	16.7%
	*VpreB1 λ5* vector	93.1%	6.9%
**Experiment 3** 5 days w/o IL-7	Control vector	89.6%	10.4%
	*VpreB1 λ5* vector	94.6%	5.4%

IL-7 dependent pro-B cells were transduced with *Bcl2* and either a *VpreB1-IRES-λ5-EGFP* or an empty *EGFP* retroviral expression construct. B cell maturation was induced by culturing the transduced pro/pre-B cells without IL-7 for 5 or 7 days, as schematized in Figure 7. The maturation state of the B cells was determined by staining for surface κLC by flow cytometry. The table shows the percentage of more mature κLC positive and less mature κLC negative cells within the *VpreB1-IRES-λ5-EGFP* transduced or mock-transduced EGFP positive B cells.

## Discussion

Aiolos/OBF-1 double-deficient mice show a developmental arrest at the transition from small to immature B cells. This block, at the small pre-BII cell subset, differs in several aspects from the phenotypes previously observed in Btk, SLP65, plcγ and Irf4/Irf8 mutant mice whose development is arrested at the large pre-BII cell stage [39], [52]–[55]. For example, pre-BII cells deficient in Irf4/Irf8 fail to exit the cell cycle, and have elevated pre-BCR on their surface [39], unlike *Aiolos^−/−^/OBF-1^−/−^* preB cells. It is worth noting that the *Irf4* and *8* genes are equally expressed in the pre-BII cells of all the mice examined here (data not shown). A block at the small pre-BII cell subset has also been observed in κ/λ double-mutant mice [56]. Although we observed a significant reduction of secondary *κ* or *λ* light chain rearrangement in *Aiolos/OBF-1* double deficient pre-BII cells, most primary *κ* light chain rearrangements and κ expression are normal in these mice. Thus, loss of light chain expression can not explain the defect in B cell maturation seen in Aiolos/OBF double-mutant mice. Gene expression analysis of the pre-BII cell stage of all four genotypes identified genes regulated by Aiolos and/or OBF-1 and some of the previously identified target genes [36] were confirmed. No major transcriptional changes were observed for any of the commonly known components of the BCR signaling pathways (*Btk*, *SLP65*, *Syk*, *Lyn*, *PKCβ*, data not shown). Thus, a function for OBF-1 in regulating *Btk* transcription in pre-B cells [57] could not be confirmed here (data not shown).

Two recent studies have described Aiolos together with Ikaros as regulators of SLC repression [38], [58]. Our analysis confirmed these observations and also additionally demonstrated an involvement of OBF-1 in the regulation of the genes encoding the SLC. ChIP experiments revealed that OBF-1 binds to three sites within the *λ5* promoter region and thereby can directly influence the regulation of the *VpreB/λ5* locus. In fact, OBF-1 deficient pre-BI cells show a striking increase in *λ5* gene expression, which however does not preclude a nearly normal silencing in pre-BII cells. The two-fold increase of *λ5* mRNA expression in OBF-1 deficient pre-BI cells indicates that at this stage OBF-1 may act as a repressor of *λ5* transcription. All three OBF-1 binding sites that we identified are located in a region upstream of the *λ5* gene that has been recently described as a locus control region. This region is highly H3-H4 dimethylated, H3 acetylated and is a target of transcription factor recruitement (PolII, transcription factor IID complex, E2A, PU.1) in pro/pre B cells [47]. Although OBF-1 has mostly been considered to be a coactivator of Oct1 or Oct2 dependent transcription, some evidence suggests that it may also contribute to transcriptional repression [59]–[61]. So far, it has not been established whether OBF1 binds to the *λ5* promoter together with Oct1 or Oct2, and whether the observed repression may need additional corepressors. However, binding of OBF-1 together with potential corepressors could interfere with the recruitment of essential transcription factors, thus decreasing transcriptional activity of *VpreB1* and *λ5*. Another possibility is that OBF-1 is not acting as a repressor, but that in its absence another, stronger, transcriptional activator can bind and lead to elevated *λ5* expression.

It has been proposed that Ikaros, and not Aiolos is the essential regulator of the *VpreB/λ5* locus [45], [62]. Both Ikaros and Aiolos can bind to two adjacent Ik1 and Ik2 sites proximal to the *λ5* transcription start site. Mutation of the more distal Ik2 site resulted in an increased expression of a *λ5* transgene in activated B cells. Because the Ik1 site overlaps with an EBF binding site within the *λ5* promoter and because the relative amounts of EBF and E2A have an effect on *λ5* transcription, it was proposed that Ikaros mediates silencing of *λ5* by competing with EBF [63]. Two very recent studies [38], [58] have demonstrated that Ikaros can counteract the effect of EBF on *λ5* expression, thereby promoting a balanced *λ5* expression at the pre-BI cell stage. Furthermore, it was found that Aiolos is likely to be the critical factor for silencing the *VpreB/λ5* locus during early B cell development. The results presented here confirm and extend the observations of these studies and additionally show that OBF-1 also contributes to the full silencing of the *λ5* locus.

Recently Parker et al. [34] have shown that silencing of this locus is accompanied by sequential repositioning of *VpreB/λ5* alleles to the nuclear periphery and to pericentromeric heterochromatic clusters. Our DNA FISH analysis shows that silencing of *VpreB/λ5* expression and repositioning of the locus within the nucleus during early B cell development is dependent on Aiolos and also on OBF-1. Surprisingly, Aiolos deficient pre-BI cells already exhibit an altered localization of the *VpreB/λ5* locus within the nucleus, although at this stage no change in *λ5* expression is seen. In contrast, OBF-1 deficient pre-BI cells show a two-fold elevated *λ5* expression, which also coincides with an altered sub-nuclear localization of *VpreB/λ5* alleles, but less pronounced than in Aiolos deficient cells. Our observations suggest that Aiolos and OBF-1 Aiolos and OBF-1 contribute by independent and different molecular mechanisms to silencing of the *VpreB/λ5* locus.

Previous studies have demonstrated that Ikaros binds to heterochromatin through multiple high affinity binding sites [49], [64]. These studies also suggested that Ikaros could directly recruit different gene loci to heterochromatic sites. An Aiolos/Ikaros heterodimer that binds to the *λ5* promoter region could target the *λ5* locus to such high affinity binding sites within the pericentromeric heterochromatic clusters.

The elevated *VpreB/λ5* expression observed in *OBF-1^−/−^* pre-BI cells is important, as in absence of OBF-1 and Aiolos not only is the silencing impaired, but the elevated expression is sustained through early B cell development. At later stages Aiolos and OBF-1 appear dispensable, as in mature B cells from *Aiolos^−/−^* or *Aiolos^−/−^/OBF-1^−/−^* mice the centromeric repositioning of the *VpreB/λ5* alleles is very similar to the repositioning seen in immature B cells from wild type mice (JAS *et al.*, data not shown). Furthermore, in splenic B cells of *Aiolos^−/−^* or *Aiolos^−/−^/OBF-1^−/−^* mice, transcription of the *VpreB/λ5* locus is completely switched off, as in wild type mice. This indicates that at the transition from small pre-BII cell to immature cells an alternative mechanism exists to silence expression of the locus. This Aiolos and OBF-1 independent silencing pathway could be mediated through binding of Ikaros homodimers at the *λ5* promoter. The complete silencing of the *VpreB/λ5* locus observed in splenic B cells of double-deficient mice also indicates that silencing is a prerequisite for immature B cells to exit from the bone marrow compartment. Other genes that are also deregulated in the bone marrow of mutant mice, but that presumably do not affect the pre-B to immature transition, for example *Gelsolin*, are still deregulated and increased in their expression in the splenic B cells from Aiolos/OBF-1 double deficient mice. In agreement with this, Gelsolin deficient mice [65] show a normal B cell development in the bone marrow and periphery (AK et al., data not shown). An alternative explanation could also be that splenic mature B cells lack positive factors that are important for *λ5* transcription. However, based on different experimental evidences, we propose that sustained elevated expression of *VpreB/λ5* interferes with the normal transition from pre-BII to immature B cells. The data presented here show that high expression of *VpreB1* and *λ5* in IL-7 dependent pre-B cells interferes with their maturation into κ light chain expressing cells upon IL-7 removal. These findings are in agreement with a recent study demonstrating that altering the level of VpreB2 expression impairs early B cell development [66]. Furthermore, the analysis of transgenic mice having a deregulated expression of the pre-TCR also supports, by analogy, the hypothesis that strong continuous expression of the pre-BCR can block B cell development [67], [68]. Finally, two very recent studies also support and confirm our results [69], [70]: In particular the analysis of *VpreB1/λ5* double transgenic mice by Van Loo *et al.* [69] revealed a reduction of immature B cells expressing high κLC and a block in early B cell development.

Although *Aiolos^−/−^* or *Aiolos^−/−^/OBF-1^−/−^* mice are both impaired in *λ5* transcriptional silencing, only the double-deficient mice have a block in the transition from pre-BII cells to immature B cells. *VpreB1/λ5* mRNA expression levels are up to 10-fold increased in small pre-BII cells, however this expression level could be below the threshold needed to elicit the phenotype at this stage. The additional increase in expression due to the loss of OBF-1 may be necessary to reach this threshold. A dosage effect has been observed in the case of the pre-TCR; a block in T cell development was detected only in mice with a high copy number and corresponding expression level of the *pTα* transgene [67]. In addition, high expression of the SLC in the small pre-BII cell subset could interfere with the positive and negative selection which occurs at the transition from small pre-BII to immature B cells. Failure of positive or negative selection activates receptor editing and secondary light chain rearrangements in immature B cells [71], [72]. Similarly, Van Loo et al. observed an increase in λLC rearrangements in *VpreB1/λ5* double transgenic B cell [69]. However, the reduction of *Vκ-Jκ5* and *λ* light chain rearrangements in Aiolos/OBF-1 double-mutant pre-BII cells suggests that the rearrangement is delayed, leading to a reduction in secondary rearrangements to *Vκ-Jκ5* and the *λ* light chain.

In summary: through this study, we are able to confirm the role of Aiolos as a regulator of *VpreB/λ5* silencing. Moreover, we could demonstrate that the developmentally regulated nuclear repositioning of the *VpreB/λ5* locus is largely dependent on Aiolos. In addition, we could identify a novel role for OBF-1 in the regulation of *VpreB/λ5* expression, as well as for the nuclear repositioning of the locus. Our findings suggest that Aiolos and OBF-1 are likely to have independent and different molecular functions in the regulation of *VpreB/λ5* expression. The combined loss of Aiolos and OBF-1 ultimately leads to a block of early B cell development at the transition from pre-BII to immature B cells.

## Supporting Information

Figure S1 Sustained cytoplasmic *Gelsolin* mRNA expression in double-deficient splenic mature B cells. (A) Gene expression profiles in IgD^+^ IgM^low^ B220^+^ mature splenic B cells were determined by MOE430a Affymetrix GeneChip; for each genotype two RNA samples were prepared from independent pools of mice and microarray analysis was done in duplicate. Figures show raw Affymetrix expression score after array normalization for *VprB1*, *VpreB2*, *λ5*, *Gelsolin* and *OBF-1*. (B) Cytoplasmic Gelsolin expression in small pre-BII and mature splenic B c ells was measured by real time RT-PCR. The real time RT-PCR assays were normalized to the *RNA polymerase II*.
(0.54 MB TIF)
