# Supplementary figures and images for: Silencing and Nuclear Repositioning of the λ5 Gene Locus at the Pre-B Cell Stage Requires Aiolos and OBF-1
Source: PLoS One. 2008 Oct 30;3(10):e3568. doi: 10.1371/journal.pone.0003568 (PMC2571989; doi:10.1371/journal.pone.0003568)

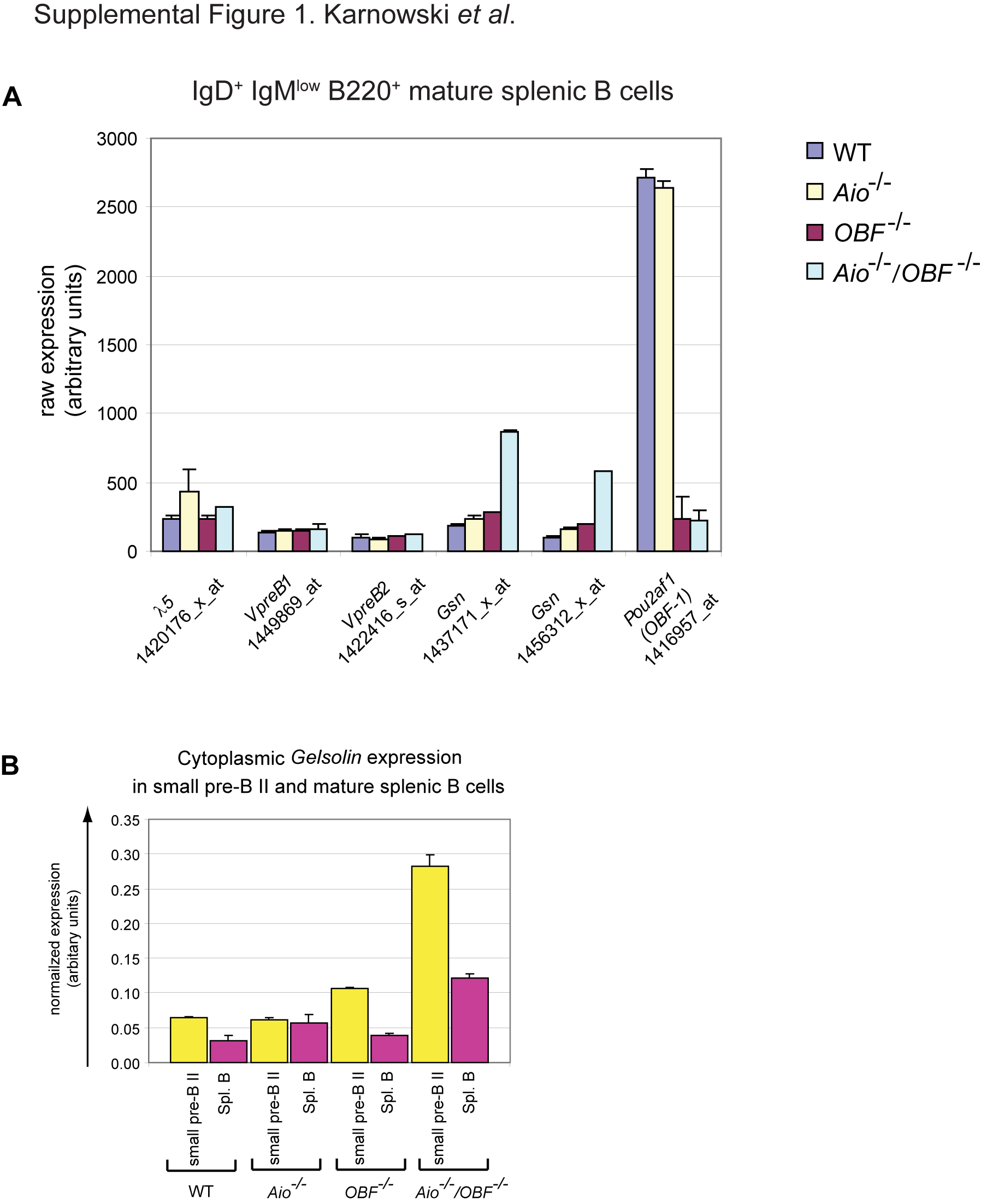

Supplement: Figure S1 — Sustained cytoplasmic Gelsolin mRNA expression in double-deficient splenic mature B cells. (A) Gene expression profiles in IgD+ IgMlow B220+ mature splenic B cells were determined by MOE430a Affymetrix GeneChip; for each genotype two RNA samples were prepared from independent pools of mice and microarray analysis was done in duplicate. Figures show raw Affymetrix expression score after array normalization for VprB1, VpreB2, λ5, Gelsolin and OBF-1. (B) Cytoplasmic Gelsolin expression in small pre-BII and mature splenic B c ells was measured by real time RT-PCR. The real time RT-PCR assays were normalized to the RNA polymerase II. (0.54 MB TIF) [file pone.0003568.s001.tif]
